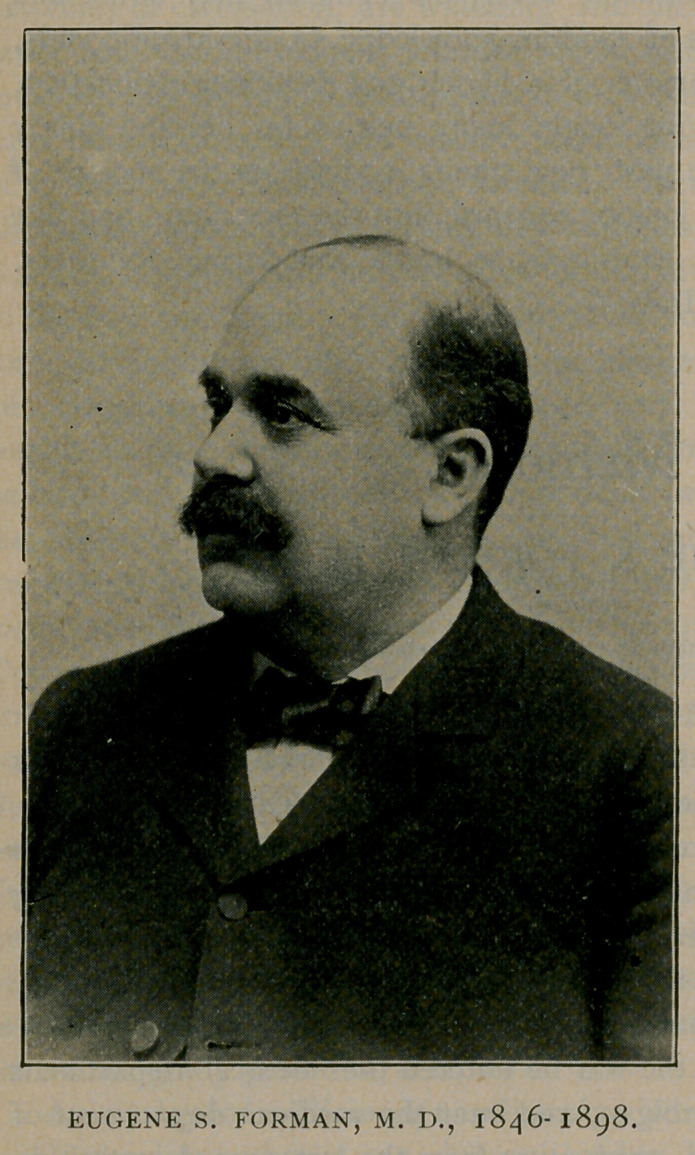# Eugene S. Forman, M. D., Auburn, N. Y.

**Published:** 1899-07

**Authors:** A. L. Hall

**Affiliations:** Fair Haven, N. Y.


### Memorial.

### EUGENE S. FORMAN, M. D.,

Auburn, N. Y.

Born October 22, 1846; died September 13, 1898.

By A. L. HALL, M. D., Fair Haven, N. Y.

THE subject of this sketch was for many years a member of this society, and at the time of his death, one of its honored vice-presidents. He was born in Cato, N. Y. His early education was obtained in the common schools and at Falley Seminary. During the Civil War he enlisted in the 3d New York Volunteer Infantry and his experience was that of every other member of a regiment, famed in the annals of our country for its gallant and heroic achievements. At the close of the war he entered the medical department of the University of Michigan, and later the medical department of the University of Buffalo, graduating from the last named institution in 1870. He immediately entered upon the practice of his profession at Meridian, N. Y., where he remained until about fifteen years ago, when he moved to the city of Auburn.

Dr. Forman's private life was an honorable one, free from blemish, and characterised by faithfulness and integrity of purpose. His career as a physician was none the less so and his genial, courteous

1. Read at the thirty-first annual meeting of the Medical Association of Cenral New York, at Auburn, October 18, 1898.

manners and untiring devotion to the sick, found substantial reward in a large and lucrative practice. His friendship was marked for its loyalty and sympathy. He was ever mindful of the comfort and enjoyment of others, and his willingness to advise and assist was a striking characteristic. A man of practical ideas and unquestionable honesty, a careful unprejudiced thinker, having a wide knowledge of medico-legal matters, his services were often required in the determination of legal controversies. For twelve years he was health officer of Auburn, and under his able management, the sanitary condition of the city was notably improved. At the time of his death and for a number of years prior thereto, he was a member of the Auburn board of United States examining surgeons, and one of the visiting physicians to the Auburn City Hospital. The discharge of the duties of these positions was marked by his characteristic faithfulness and ability.

EUGENE S. FORMAN, M. D., 1846-1898.

He possessed the confidence of his medical colleagues, and in matters pertaining to the advancement and promotion of his profession, he was always an earnest and interested worker. For many years he was an active member of the Medical Society of the County of Cayuga, of which he had been president. He was a member of the American Medical Association and attended the meeting at Denver in June last. He was also one of the delegates from the county society to the State Medical Society and had served two years as such.

He is survived by his wife and two children—Dr. Andrew J. Forman, who will hereafter conduct his practice, and a daughter, Mrs. William C. Hamilton, of Pittsburg, Pa.

About six years ago Dr. Forman had cerebral hemorrhage involving the motor tract of the right leg from which he recovered, but which impaired his physical powers. For two weeks prior to his death, he had a mild form of intestinal catarrh, which was thought to be of no serious import, and in spite of which, he continued to attend to his professional work. On the evening of his death, he reduced a fracture of the forearm, which, probably seriously taxed the strength of his already weakened system. Later in the evening while proceeding, in response to a professional call, to enter his carriage accompanied by his faithful wife, suddenly and without warning, he sank to the ground and immediately expired. Death was ascribed to cardiac failure, superinduced by overexertion, occurring in an organ whose muscular structure was known to have been weakened by chronic degenerative changes. The funeral services were held from the family residence and interment was made in Fort Hill cemetery at Auburn. He left the memory of a noble character, worthy of our emulation.

## Figures and Tables

**Figure f1:**